# Penetration and Effectiveness of Micronized Copper in Refractory Wood Species

**DOI:** 10.1371/journal.pone.0163124

**Published:** 2016-09-20

**Authors:** Chiara Civardi, Jan Van den Bulcke, Mark Schubert, Elisabeth Michel, Maria Isabel Butron, Matthieu N. Boone, Manuel Dierick, Joris Van Acker, Peter Wick, Francis W. M. R. Schwarze

**Affiliations:** 1 ETH, Institute for Building Materials, Zürich, Switzerland; 2 Empa, Applied Wood Materials, Dübendorf/ St. Gallen, Switzerland; 3 UGCT - Woodlab-UGent, Laboratory of Wood Technology, Department of Forest and Water Management, Faculty of Bioscience Engineering, Ghent University, Ghent, Belgium; 4 Empa, Advanced Fibers, St. Gallen, Switzerland; 5 UGCT - Radiation Physics, Department of Physics and Astronomy, Proeftuinstraat 86/N12, Ghent University, 9000 Ghent, Belgium; 6 Empa, Particles-Biology Interactions, St. Gallen, Switzerland; University of South Australia, AUSTRALIA

## Abstract

The North American wood decking market mostly relies on easily treatable Southern yellow pine (SYP), which is being impregnated with micronized copper (MC) wood preservatives since 2006. These formulations are composed of copper (Cu) carbonate particles (CuCO_3_·Cu(OH)_2_), with sizes ranging from 1 nm to 250 μm, according to manufacturers. MC-treated SYP wood is protected against decay by solubilized Cu^2+^ ions and unreacted CuCO_3_·Cu(OH)_2_ particles that successively release Cu^2+^ ions (reservoir effect). The wood species used for the European wood decking market differ from the North American SYP. One of the most common species is Norway spruce wood, which is poorly treatable i.e. refractory due to the anatomical properties, like pore size and structure, and chemical composition, like pit membrane components or presence of wood extractives. Therefore, MC formulations may not suitable for refractory wood species common in the European market, despite their good performance in SYP. We evaluated the penetration effectiveness of MC azole (MCA) in easily treatable Scots pine and in refractory Norway spruce wood. We assessed the effectiveness against the Cu-tolerant wood-destroying fungus *Rhodonia placenta*. Our findings show that MCA cannot easily penetrate refractory wood species and could not confirm the presence of a reservoir effect.

## Introduction

Wood is a widely used building material and one of the reasons is its availability in various countries. This also results in a geographic-dependent decking market, as each country mostly utilizes the most accessible wood species. For instance, the North American market mostly uses SYP [1], whereas Norway spruce is the most used species in Central Europe [2].

The choice of a certain wood species for building applications results in clearly defined performance and service life, which can range from a few to many years. The specific anatomical and compositional features of wood are species-specific and cause major differences in the natural durability and the permeability to wood preservatives (or treatability), resulting in different wood species-based durability classes. According to the EN 350–2 [3] treatability is defined as the ease at which wood can be penetrated by liquids, e.g. wood preservatives. This mainly depends on the wood openings, the size and structure of tracheids, fibers, vessels, bordered- and simple pits. Due to the large size of the simple pits SYPs (*Pinus caribaea*, *Pinus echinata*, *Pinus palustris*, and *Pinus taeda*) are considered as easily treatable species, especially their sapwood [3]; whereas Norway spruce sapwood and particularly heartwood [4] are refractory to wood preservatives due to closure of the bordered pits [5, 6]. Therefore, wood preservative treatments developed for the easily treatable wood of the North American market may not be suitable for refractory wood species, like European widely used Norway spruce [2], or they may require additional treatments prior to impregnation, e.g. wood incising. This processfacilitates drying of refractory species [7], and improves the penetration and retention of preservatives [8, 9].

One of the most common and at the same time most demanding building applications for wood is for structures in contact with the soil, defined as use class 4 by the European Standard EN 335 [10]. The latter wood products have to be treated with copper (Cu)-based wood preservatives to avoid decay by soil-borne microorganisms, especially soft-rot fungi [11]. The latest advance in Cu-based wood preservation is known as “micronized copper” (MC), and consists of basic Cu carbonate (CuCO_3_·Cu(OH)_2_) particles with a size range of 1 nm-250 μm, according to manufacturers, combined with a second organic biocide that provides protection against basidiomycetes, either azole (MCA) or quaternary ammonium compounds (MCQ) [12,13]. MC reputedly has a special impregnation chemistry that differs from conventional wood preservatives [14], i.e. part of CuCO_3_·Cu(OH)_2_ immediately solubilizes during the impregnation process and is complexed by wood organic macromolecules, while another fraction does not react and acts as reservoir, i.e. Cu is solubilized afterwards [14] and provides a continuous protection against wood-destroying fungi [15].

The entire literature on Cu distribution and speciation of this wood preservative in wood deals with easily treatable SYP [15–22], Scots pine wood [23], or red pine [17], and hypotheses on the possible penetration ability of MC in refractory wood species have been proposed [24]. However, to our knowledge there are no studies available that demonstrate the benefits of MC formulations for refractory wood species, either from North America or from Europe. Thus actual data on the Cu distribution from MC-treated refractory wood, as well as on its resistance against wood-destroying basidiomycetes are missing.

We hypothesize that the properties of MC by themselves cannot guarantee a homogeneous wood preservative penetration into refractory wood species, similarly to conventional wood preservatives. In order to verify that, in this paper we assess and compare the penetration of copper from MCA in Scots pine sapwood, Norway spruce sapwood and heartwood without any prior incision of wood. The penetration of Cu was assessed directly by means of X-ray computed tomography (CT) and ion-coupled plasma-optical emission spectroscopy (ICP-OES), and by indirect methods provided by the European standard EN 113 guidelines [25]. In addition, we compared MCA penetration effectiveness with its protective effectiveness against the Cu-tolerant [26] wood-destroying fungus *Rhodonia placenta*, which we previously used to test the ionic, nano, and bulk Cu effects of MCA [27]. By using a Cu-tolerant basidiomycete we could get an insight into the mechanisms behind MC superior effectiveness compared to conventional wood preservatives, as the fungus would not immediately succumb due to the presence of Cu, even if minimal, as it would happen with soft rot fungi.

## Material and Methods

### Materials

A commercial aqueous suspension of MCA was investigated. The formulation tested here coincides with the formulation with high amount of tebuconazole MCA_HTBA we used in our previous investigation [27]. The latter study also provides a full characterization of the Cu particles in the MCA formulation. In brief, the measured particle size distribution of MCA was 104±1.7 nm with an average zeta potential of -21±0.4 mV.

Wood blocks (50 x 25 x 15 mm) were excised from Scots pine (*Pinus sylvestris* L.) sapwood, Norway spruce (*Picea abies* (L.) Karst.) heartwood and sapwood. Norway spruce sapwood and heartwood were localized by visual inspection, the heartwood was selected from an area as close as possible to the center of the trunk, while sapwood was selected from the outer region. The wood samples were pressure-treated according to the European standard EN 113 [25] with different concentrations of a commercial MCA aqueous suspension (100.00%, 2.00%, 1.60%, 1.33%, 1.07%, 0.80%, 0.00%). Three repetitions of 100.00% MCA-pressure-treated wood samples and six replicates for the diluted MCA-pressure-treated wood specimens were prepared. Small needle-shaped wood specimens (5 x 0.5 x 0.5 mm) from Scots pine sapwood, Norway spruce sapwood and heartwood were cut from the outer surface of EN 113 untreated blocks and were subsequently treated with MCA by dipping them into 100.00% MCA. No permits were required for the described study, which complied with all relevant regulations. No endangered or protected species were involved.

### Cu penetration, uptake and distribution in wood

#### Preservative retention

Preservative retention in kg/m^3^ was calculated following the guidelines from the European standard EN 113 [25] with the following formula:

$$Preservative\;retention=\frac{Solution\;uptake*Solution\;concentration}{Volume\;of\;wood\;sample}*1000$$

The solution uptake was calculated as the difference between the wood samples’ wet mass after impregnation and the oven dried (103±1°C for 18 h minimum) initial mass prior impregnation, according to the European standard EN 113 [25].

#### Quantification of Cu in wood

Cu in wood was quantified by ICP-OES (Perkin-Elmer OPTIMA 3000, detection limit: 0.005 mg/L). For Scots pine sapwood, Norway spruce sapwood and heartwood EN 113 wood blocks untreated and pressure-treated with 100% MCA were ground into sawdust. Samples from the inner wood were selected cutting the first 15 mm off of the samples off and grinding the inner surface obtained. In addition samples from the outer edge (first 15 mm) of Scots pine sapwood were collected. In this way, a penetration gradient from the easily treatable wood species could be assessed. Digestion of the samples prior to ICP-OES was conducted according to Platten et al. [28]. Three replicates of each sawdust sample were digested with 3 mL of HNO_3_ (65%) and 1 mL of H_2_O_2_ (30%) (MLS 1200 MEGA digestion system). Cu plasma standard solutions (100 mg/L) were used for calibration.

#### Visualization of Cu in wood

Before and after treatment, EN 113 wood blocks and small needle-shaped wood specimens from the three wood materials considered (Scots pine sapwood, Norway spruce sapwood and heartwood) pressure-treated with 100.00% MCA were analyzed by means of the multi-resolution micro-CT scanner Nanowood [29, 30] built at the Ghent University Centre for X-ray Tomography (Ghent University, Belgium). The EN 113 blocks were visualized to assess the overall penetration of Cu. The samples were scanned using a closed type microfocus X-ray tube at 100 kV and 80 μA, 1000 projections and 1000 ms exposure time per projection. Reconstructions were performed with the Octopus Reconstruction software package [31], a tomography reconstruction package for parallel, cone-beam and helical geometry licensed by InsideMatters (www.insidematters.be), resulting in reconstructed data with an approximated voxel pitch of 31 μm. The reconstructed volumes were analyzed using Octopus Analysis, previously known as Morpho+ [32] and also licensed by InsideMatters, to approximately visualize Cu distribution. Therefore, the reconstructed volumes were bilateral filtered to remove noise with edge preservation. Subsequently the wood was separated from the surrounding air such that further calculations were only performed within the wood block. Finally, Cu was segmented based on thresholding. The threshold level of the latter segmentation was chosen conservatively such that no wood was selected, which was based on scans of untreated wood blocks scanned with identical settings. Volumes were also rendered in 3D using VGStudio Max software.

The smaller needle-shaped specimens were used to investigate the Cu distribution in wood at the cellular level. The samples were scanned using an open type nanofocus X-ray tube at 80 kV and 45 μA, 1000 projections and 1500 ms exposure time per projection. Reconstructions were also performed with the Octopus Reconstruction software package, resulting in volumes with an approximate voxel pitch of 0.8 μm. Phase contrast effects were reduced using the Paganin method [33], also significantly improving the signal-to-noise ratio [34]. Due to the violation of the homogeneous object assumption in this method, additional smoothing around the MC is however introduced [35]. Obtaining quantitative results from these data is therefore not directly possible.

For both the low and high resolution scans, approximate detection limits on X-ray CT scans of Cu in wood were calculated, using the NIST XCOM database [36].

### Effectiveness against Cu-tolerant basidiomycetes

After drying, the 2.00%, 1.60%, 1.33%, 1.07%, 0.80%, and 0% MCA-pressure-treated wood samples were exposed for 16 weeks at 22°C and 70% RH to the Cu-tolerant wood-destroying basidiomycete *R*. *placenta* isolate 45 from the Empa culture collection. Test procedures were performed according to the European standard EN 113 [25]. After incubation, wood blocks were removed from the culture vessels, brushed free of mycelium and oven dried at 103±1°C for a minimum of 18 h. The percentage of mass loss was calculated from the dry weight before and after the test.

Some of the results from MCA-pressure-treated Scots pine wood were formerly published in a previous study [27], and we integrated it here to provide a complete overview on the effectiveness of MCA in different wood species.

### Statistical analysis

Preservative retention data were log-transformed and data expressed as percentages (Cu amounts in wood and wood mass losses) were arcsine-transformed prior to analysis (ANOVA) and back-transformed to numerical values for visualization. Means were separated using Tukey’s-HSD (Honestly Significant Difference) test at significance level p<0.05. The statistical package used for all analyses was SPSS^®^ (Version 17.0, SPSS Inc., Chicago, IL, USA).

## Results

### Cu penetration, uptake and distribution in wood

#### Preservative retention

**Fig 1:**
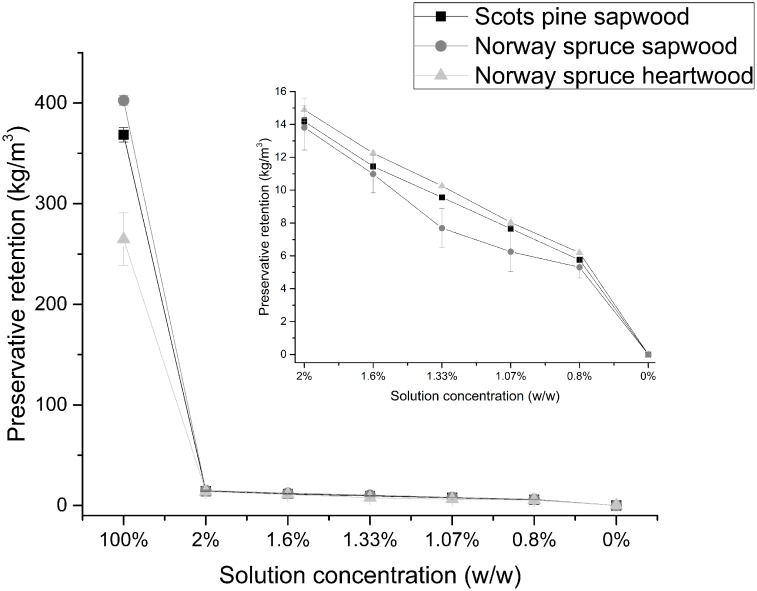
Preservative retention in Scots pine sapwood, Norway spruce sapwood, and Norway spruce heartwood calculated according to the EN 113 guidelines [25]. Data are represented as mean ± standard deviation of three replicates. Shared letters indicate treatments that were not significantly different, different letters denote significant differences in treatments after the Tukey’s HSD test.

According to the European standard EN 113 [25], we gathered indications on the expected MCA retention in easily treatable Scots pine sapwood and refractory Norway spruce sapwood and heartwood (Fig 1).

The three wood materials considered share the same preservative retention pattern (p-value = 0.137) across all diluted MCA-pressure-treated wood samples (2.00%, 1.60%, 1.33%, 1.07%, 0.80%), which decreases with the MCA concentration applied. Therefore, comparable amounts of Cu could be expected in different wood species pressure-treated with the same MCA concentration, independently of their permeability. This pattern was not applicable to the 100% MCA-pressure-treated wood samples, where the preservative retentions were certainly lower, even though the expected amount of Cu was higher. Moreover, a difference between the refractory Norway spruce heartwood and the more accessible Norway spruce sapwood and the easily treatable Scots pine sapwood was visible.

#### Quantification of Cu in wood

**Fig 2:**
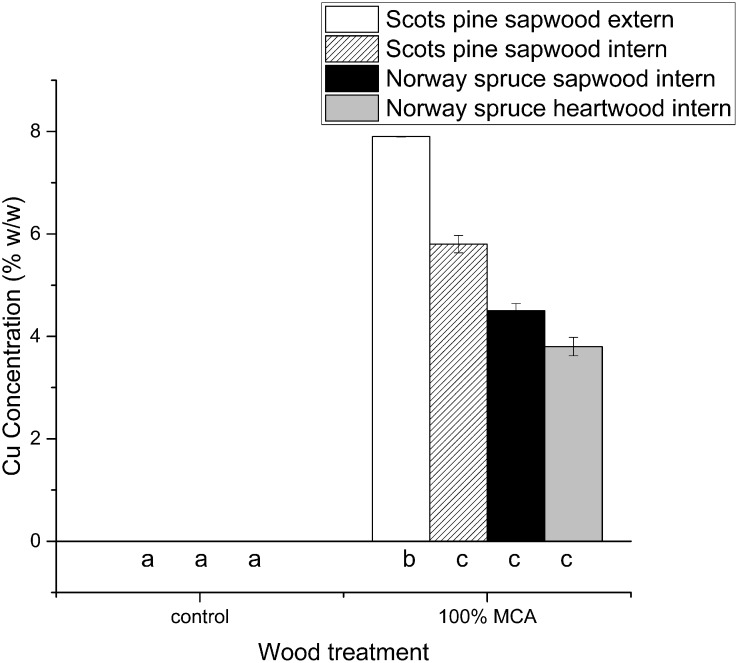
Percentage of Cu in Scots pine sapwood (inner and outer surface), Norway spruce sapwood (inner), and Norway spruce heartwood (inner) measured by ion-coupled plasma-optical emission spectroscopy (ICP-OES). Data are represented as mean ± standard deviation of three repetitions. Shared letters indicate treatments that were not significantly different, different letters denote significant differences in treatments after the Tukey’s HSD test.

We quantified the amount of Cu in both easily treatable Scots pine (sapwood) and refractory Norway spruce (sapwood and heartwood). Fig 2 summarizes the weight percentages of Cu detected by ICP-OES in untreated and 100.00% MCA-pressure-treated Scots pine sapwood (inner and outer regions), Norway spruce sapwood (inner), and Norway spruce heartwood (inner).

When untreated, both Scots pine and Norway spruce wood contain comparable (p-value = 1) negligible amounts of Cu (below detection limit). In 100.00% MCA-pressure-treated wood samples, Scots pine sapwood (outer regions) had the highest amount of Cu. This percentage decreased in the interior of Scots pine sapwood, but the values remained higher than in Norway spruce sapwood or heartwood. The latter contained the lowest Cu amount, thus Norway spruce heartwood was the most refractory wood treated here. The Tukey’s HSD test indicates that the Cu amount in 100.00% MCA-pressure-treated Norway spruce sapwood was not significantly different from the one in 100.00% MCA-pressure-treated Norway spruce heartwood (p value = 0.908) and the interior part of Scots pine sapwood (p-value = 0.489).

#### Visualization of Cu in wood

X-ray CT and subsequent analysis allowed qualitative and semi-quantitative determination of Cu in MCA-pressure-treated Scots pine sapwood, Norway spruce sapwood, and Norway spruce heartwood. Thresholding of the EN 113 wood blocks was performed to distinguish Cu in the tomographic reconstructions. The same conservative threshold was used for all EN113 wood samples.

**Fig 3:**
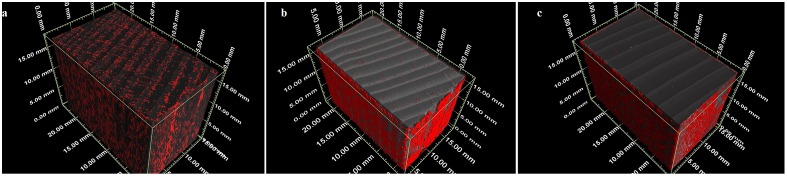
Three-dimensional reconstruction of Cu penetration (red) in (a) Scots pine sapwood, (b) Norway spruce sapwood, and (c) Norway spruce heartwood.

In Fig 3 the threshold-based Cu penetrations (red) in the three wood materials considered are visible. The Cu thresholding on the 31 μm resolution scans of the 100.00% MCA-pressure-treated Norway spruce samples accounted for only 1% (heartwood) or up to 2% (sapwood) of the volume, which was significantly lower than the percentages detected by ICP-OES.

In Scots pine and Norway spruce sapwood blocks it was clearly visible that the penetration of Cu occurred predominantly via resin canals and was more abundant in the latewood. In Scots pine Cu was also present within the rays. It was furthermore calculated that the detection limit of Cu would be of the order of 0.1 μg per voxel for the low resolution scans.

**Fig 4:**
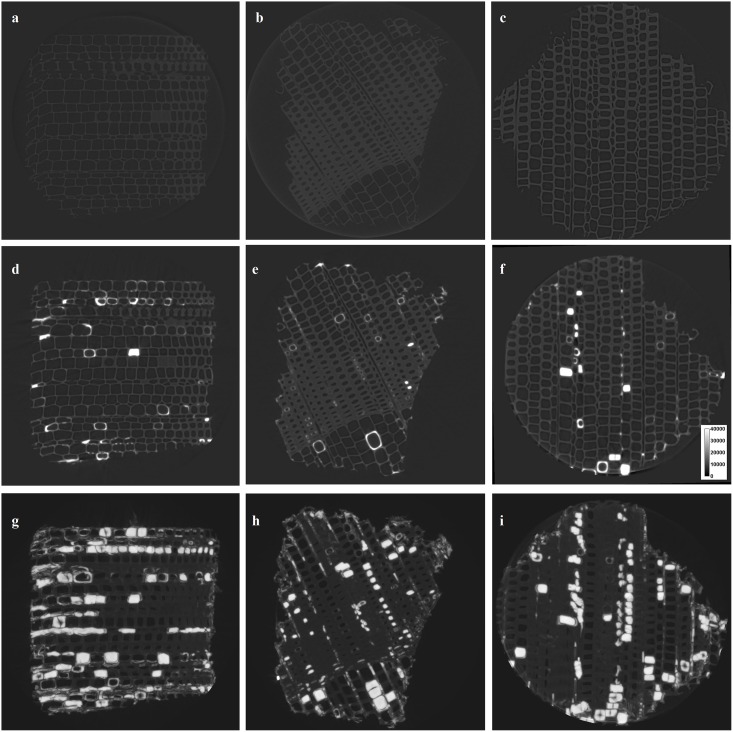
Reconstructed slices before (above), after (middle) MCA-pressure-treatment, and maximum intensity projections after MCA-pressure-treatment (below) of Scots pine sapwood (a, d, g), Norway spruce sapwood (b, e, h), and Norway spruce heartwood (c, f, i). The brighter spots indicate areas containing high-density elements (Cu).

At the cellular level, the greyscale patterns in Scots pine and Norway spruce wood coincide, with bright spots (high-density Cu) around the cell wall or filling the cell lumina, as indicated in Fig 4. In the three wood materials considered no major difference in the Cu uptake by ray parenchyma and ray tracheids was observed. The localization of Cu in the wood ultrastructure and the form of Cu, however, remain uncertain. In particular, even with the maximum intensity projections, it is unclear whether Cu is into or on the cell wall, and if ions, nanoparticles, or aggregates/agglomerates are responsible for the larger bright areas within the cell wall. It was furthermore calculated that the detection limit of Cu would be of the order of 2 pg per voxel for the high resolution scans.

### Effectiveness against Cu-tolerant basidiomycetes

**Fig 5:**
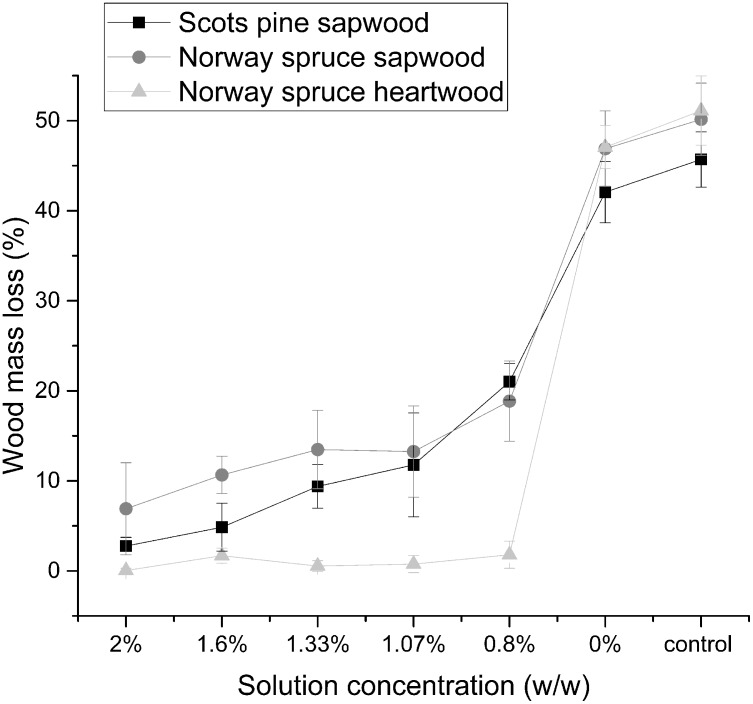
Assessment of micronized Cu azole (MCA) concentrations against *R*. *placenta* 45 and associated mass losses in Scots pine sapwood, Norway spruce sapwood, and Norway spruce heartwood. Data are represented as mean ± standard deviation of six replicates. Plain font was used for Scots pine sapwood, underline for Norway spruce sapwood, and italics for Norway spruce heartwood. Shared letters within the same wood type indicate treatments that were not significantly different, different letters denote significant differences in treatments after the Tukey’s HSD test.

We assessed if the difference in Cu penetration in the three MCA-pressure-treated wood materials considered affected the wood protection effectiveness against the Cu-tolerant fungus *R*. *placenta* 45. Wood mass losses for the different wood species and MCA concentrations are reported in Fig 5.

Tukey’s HSD test showed no differences between the mass losses from 0.00% MCA and control wood samples for each of the three wood materials considered. Among the different wood species, the mass losses in control samples were slightly lower for Scots pine sapwood (p-value < 0.001), while they were equivalent for Norway spruce sapwood and heartwood (p-value = 0.456). Differences between the sapwood and heartwood from Norway spruce were recorded after treatment with MCA (p-value < 0.001). All MCA concentrations prevented *R*. *placenta* 45 from colonizing Norway spruce heartwood, with mass losses well below 3.0%. On the contrary, despite a significant reduction in mass losses when compared to the 0.00% MCA and control, even concentrations of 1.60% MCA were not sufficient to protect Norway spruce sapwood from degradation (mass loss: 10.7±2.1%). At concentrations of 2.00% MCA decay results were more variable, with recorded mass losses between 0.3% and 13.5%. Mass losses from Scots pine control samples were not comparable to the ones from Norway spruce sapwood and heartwood (p-value < 0.001). Scots pine MCA-pressure-treated samples appeared significantly decayed below concentrations of 2.00% MCA (above 3%), while the latter caused mass losses of 2.75±0.975%.

## Discussion

The aim of this study was to assess if MC could penetrate refractory wood species without pre-treatment, i.e. incising, and consequently provide an added value than conventional wood preservatives. We compared the pressure-treatment penetration effectiveness of Cu from an MCA formulation in easily treatable Scots pine sapwood and refractory Norway spruce sapwood and heartwood. The comparison was carried out using three different techniques: the indirect calculation of wood preservative retention after impregnation, as indicated by the EN 113 guidelines [25], the quantification of Cu by ICP-OES, and the density-based greyscale thresholding on X-ray CT reconstructions. We also aimed to correlate MCA penetration with protective effectiveness against the wood-destroying fungus *R*. *placenta* 45.

The nature of the EN 113 preservative retention formula [25] does not consider the treatability of the wood species. In the present study this resulted in an equal amount of expected MCA penetrating in the Scots pine and Norway spruce wood blocks at a given MCA concentration, and a linear correlation between the MCA concentration and the preservative retention, independent of the wood species. This calculation appears to diverge from the directly measured amount of Cu in the three wood materials considered. The ICP-OES analysis revealed that the background level of Cu present in untreated wood is negligible, as its concentrations in both Scots pine and Norway spruce (heartwood and sapwood) were below the instrument’s detection limit. Therefore, the amount of Cu detected in MCA-pressure-treated wood can be attributed solely to the wood preservative. Our results from the ICP-OES measurements on MCA-pressure-treated wood indicate that Cu was more abundant in Scots pine sapwood, especially on the surface, and only half of the Cu percentage found in Scots pine was detected in Norway spruce heartwood, the most refractory wood in this study. These results clearly showed that the amount of Cu penetrating into the wood heavily depends on the wood species and on the presence of sapwood (more accessible) or heartwood (less accessible) [4]. In addition, X-ray CT scanning and subsequent analysis enabled Cu distribution visualization in wood based on thresholding of the images. It should be noted that this results in semi-quantitative data, since it is not trivial at all to derive quantitative data from X-ray CT scans. Furthermore, comparison with other methods for quantification of Cu in wood, is not straightforward as well since the thresholding applied, results in a percentage of voxels containing Cu, and does not relate to the exact amount of Cu present within those voxels. Although regions containing very low amounts of Cu could be overlooked, a detection limit of approximately 0.1 μg is small enough such that, if present, it would be visible.

For Scots pine sapwood 7% of the Scots pine wood block volume was found to be Cu. We applied the same thresholding on Norway spruce sapwood and heartwood reconstructions. While Cu was rather homogeneous across the whole Scots pine wood section, similarly to what is observed in other easily treatable species [16–23], in Norway spruce wood it was mostly located on the surface. In accordance with Evans et al. [18] and in good agreement with findings on western hemlock by Xue et al. [37], Cu was more abundant in the latewood and mainly distributed in rays and resin canals. Further, the Cu distribution pattern coincides with the one highlighted from MC by Evans et al. [16], which differs from the Cu distribution pattern from conventional amine Cu wood preservatives [16]. The threshold of Norway spruce sapwood and heartwood resulted in lower Cu percentages amounts than those detected by ICP-OES. This indicates that most of Cu is not present as particles, aggregates or agglomerates with size detectable at 31 μm, i.e. the resolution of the scans. This was confirmed by the scans at higher resolution, where Cu was visible at the cell wall level. Although regions containing very low amounts of Cu could be overlooked, a detection limit of approximately 2 pg is small enough such that, if present, it would be visible. The submicron resolution, however, did not allow to precisely locate Cu in the cell wall, i.e. if Cu diffuses into (cell-wall treatment) or only onto it (cell-lumen treatment). This issue has previously been hypothesized [38], and the difference is critical for wood protection because wood cell-wall treatments are certainly more effective against white and soft rot fungi [39, 40] and also more resistant to leaching [41]. In addition, it was not possible to determine if the Cu present was solubilized or available as unreacted -single or aggregated/agglomerated- particles responsible for a reservoir effect. Cu appeared equally distributed in ray parenchyma and ray tracheids, similarly to what Olsson et al. [42] observed. This confirms that despite the innovative impregnation chemistry of MC, the wood preservative fluid flow is still subject to the same resistance, i.e. the pits between ray parenchyma and tracheids. In fact, while in easily treatable Scots pine, these cross-field pits are fenestrate and with a large thin membrane, in Norway spruce they are of the piceoid type, with smaller membrane and smaller dimensions [43, 44].

The effectiveness of MCA against the Cu-tolerant fungus *R*. *placenta* greatly differed for Scots pine sapwood, Norway spruce sapwood and heartwood. Despite the valid performance of MCA-pressure-treated Norway spruce heartwood even at the lowest MCA concentration (0.80%), the protection of Scots pine wood appeared to be more difficult. The different patterns cannot be explained by differences in fungal virulence or wood natural durability, as the mass losses for Scots pine sapwood control samples were slightly lower than those of Norway spruce sapwood or heartwood. One explanation may be related to the Cu-tolerance of *R*. *placenta* and the interactions between Cu and tebuconazole within the MCA formulation. As previously demonstrated [27], tebuconazole plays a major role as active ingredient against *R*. *placenta*, however sub-lethal concentrations of Cu in tebuconazole-amended media can actually stimulate the growth of the fungus. In the present study Norway spruce heartwood samples contained the lowest amount of Cu, and most of it was located at the surface. The same is likely to apply for the co-biocide tebuconazole, whose low amount could still suffice to exert an antifungal effect. Hence the fungus has almost no Cu that would help it surviving in a tebuconazole-amended media. In Norway spruce sapwood the amount of Cu measured by ICP-OES increased slightly, providing *R*. *placenta* the conditions to survive despite the presence of tebuconazole. Finally, in Scots pine wood the amounts of Cu were even higher, providing even more resources, until at high MCA concentrations Cu becomes lethal together with tebuconazole (threshold concentration). Another possible explanation involves the amount of reacted Cu. Most of Cu contained in Norway spruce heartwood is likely to be in the form of fine particles (below 31 μm) or ions, as indicated by the low resolution X-ray CT scan and the thresholding coupled with the ICP-OES analysis, whereas Cu appears in larger clusters in Norway spruce sapwood, and even larger in Scots pine wood. This could result in a reduction in the ratio between solubilized bioavailable Cu^2+^ and unreacted CuCO_3_·Cu(OH)_2_ in more easily treatable wood. Therefore, in the short-term the MC reservoir effect, i.e. unreacted CuCO_3_·Cu(OH)_2_ particles slowly solubilizing in wood, may be counterproductive. In fact, the short-term nature of the EN 113 studies, which last only 16 weeks, would not allow to assess the long-term protection provided by a reservoir effect, which may be visible after several months or years. Despite their different nature, the two possible explanations, i.e. Cu-tebuconazole interaction or reacted-unreacted Cu ratio, share a common conclusion: the CuCO_3_·Cu(OH)_2_ particles in MCA formulations do not contribute to a better short-term performance because they either support the growth of the wood-destroying fungus, or are not bioavailable and cannot exert an antifungal effect.

In the long-term, further mechanisms beside the reservoir effect should be considered. Cu from MCA-pressure-treated Norway spruce can be released in the environment, similarly to what is observed for MCA-pressure-treated SYP [28], thus the environmental impact should be considered.

In conclusion, our hypothesis that MC cannot readily penetrate refractory wood species, which are commonly used in Central Europe, was confirmed. Therefore, from a wood penetration perspective, MC performance is comparable to conventional wood preservatives, and the adoption of MC for refractory wood species in the European market would still require pretreatment such as incising. However the MCA formulations succeeded in protecting refractory wood species against *R*. *placenta*, and the treated refractory wood was destroyed less than easily treatable MCA-pressure-treated wood. Nevertheless in the short-term CuCO_3_·Cu(OH)_2_ particles do not provide an added value for the wood preservative formulation. Future studies should focus on MC’s Cu speciation in wood and its interaction with wood ultrastructure. In this way, the presence of a reservoir effect, of cell-wall or cell-lumen treatments, and the basis of MCA effectiveness could be thoroughly understood.
